# Comment on Kao et al. Pulmonary Fat Embolism Following Liposuction and Fat Grafting: A Review of Published Cases. *Healthcare* 2023, *11*, 1391

**DOI:** 10.3390/healthcare12131326

**Published:** 2024-07-03

**Authors:** Pouria Chaghamirzayi

**Affiliations:** Clinical Research Development Unit of Shahid Madani Hospital, Alborz University of Medical Science, Karaj 3143744693, Iran; pouriachaghamirzayi@yahoo.com

I recently read the review article entitled “Pulmonary Fat Embolism Following Liposuction and Fat Grafting: A Review of Published Cases” by Kao et al. [1], which was published in The Healthcare (Basel) Journal (2023;11(10)). I wish to make some constructive comments and corrections regarding certain aspects of the article.

In Table 1, which provides an overview of the reviewed studies and patients’ characteristics, a few discrepancies need to be addressed. The authors mentioned the reports of Foula et al. [2] and Foula et al. [3] twice in the table, once for a 2019 study from Saudi Arabia and once for a 2022 study from Egypt. It should be noted that the 2019 citation is a poster presentation derived from the same study, and it represents the same patient. Therefore, combining these two citations into a single entry would be more accurate (Table 1 revised).

Additionally, the study of Coronado-Malagón M et al. [4] should not be included in this review based on the inclusion criteria. The patient in this case report did not undergo liposuction; only a soft tissue filler was injected. Therefore, this case report is not eligible for inclusion in a review focused on pulmonary fat embolism following liposuction and fat grafting (Table 1 revised). 

Regarding the “Surgery” section of Table 1, the authors stated that the procedures in the studies of Laub and Laub [5], Foula et al. [2,3], Erba et al. [6], Saon et al. [7], Kadar et al. [8] and Pham et al. [9] involved “liposuction and fat grafting”. These studies did not include “fat grafting”. For example, the study by Foula et al. [2,3] describes a case wherein cardiac arrest occurred before lipofilling, and the other studies involved procedures that did not include fat grafting. The surgery section is revised and shown in the revised Table 1.

In the “Mortality” section, all patients are marked as “Alive”, which is inaccurate. The revised form of the mortality section of Table 1 is shown in the revised Table 1.

Considering these corrections, the total number of studies in the article changed to 36, and the number of patients to 38. Liposuction was performed in all patients, and fat grafting was performed in 13/38 (34.3%) of patients. Regarding mortality rate, the outcomes of two patients were not described in the two studies [10,11] and 12 patients died. Therefore, the corrected mortality rate is 12/36 (33.3%).

## Figures and Tables

**Table 1:** (revised). Reviewed studies and patients’ characteristics.

Year, Author *	Country	Sex	Age	Surgery	Cardiac Arrest	Mechanical Ventilation	Mortality
1983, Hunter GR [15]	US	F	37	Liposuction	No	Unknown	Alive
1986, Christman KD [16]	US	F	56	Liposuction	Yes	Yes	Dead
1988, Ross RM [17]	US	F	44	Liposuction	No	Yes	Alive
1990, Boezaart AP [18]	South Africa	F	39	Liposuction	No	Yes	Alive
1990, Laub Jr DR [19]	US	F	51	Liposuction	No	No	Alive
1997, Currie I [20]	Canada	F	69	Liposuction, fat grafting	Yes	Yes	Dead
1998, Fourme T [21]	France	F	29	Liposuction	No	No	Alive
1999, Folador JC [22]	Brazil	F	40	Liposuction	No	No	Alive
1999, Scroggins C [23]	US	F	54	Liposuction	No	Yes	Alive
2002, Platt MS-1 [14]	US	F	82	Liposuction	Yes	Yes	Dead
2002, Platt MS-2 [14]	US	M	50	Liposuction	Yes	Yes	Dead
2006, Rothmann C [24]	France	F	24	Liposuction	No	Yes	Alive
2007, Wessman DE [25]	US	M	31	Liposuction	No	No	Alive
2008, Costa AN [26]	Brazil	M	53	Liposuction, fat grafting	No	Yes	Alive
2011, Erba P [27]	Switzerland	F	46	Liposuction	No	Yes	Alive
2011, Gleeson CM [28]	UK	F	37	Liposuction, fat grafting	Yes	Yes	Dead
2012, Shiffman MA [29]	US	F	40	Liposuction, fat grafting	Yes	Yes	Dead
2013, Zeidman M [30]	US	F	24	Liposuction	No	Yes	Alive
2014, Cohen L [31]	US	F	58	Liposuction	Unknown	Unknown	Unknown
2014, Hostiuc S [32]	Romania	F	56	Liposuction	Yes	Yes	Dead
2015, Astarita DC [33]	US	F	42	Liposuction, fat grafting	Yes	Yes	Dead
2015, Byeon SW [34]	Korea	M	21	Liposuction	No	Yes	Alive
2015, Cárdenas-Camarena L [35]	Colombia	F	37	Liposuction, fat grafting	Yes	Yes	Dead
2015, Fu X [36]	China	F	30	Liposuction, fat grafting	No	No	Alive
2015, Vidua RK [37]	India	F	39	Liposuction	Yes	No	Dead
2016, Souza RL [12]	Brazil	F	42	Liposuction, fat grafting	No	Yes	Dead
2017, Ali A [38]	UK	F	45	Liposuction	No	Yes	Alive
2017, Sasaki Y [39]	Japan	F	29	Liposuction	No	Yes	Alive
2017, Zilg B [40]	Sweden	M	31	Liposuction, fat grafting	Yes	Yes	Dead
2019, Peña W [41]	Mexico	F	41	Liposuction, fat grafting	Yes	Yes	Alive
2019, Saon MD [42]	US	F	52	Liposuction	No	No	Alive
2020, Recinos S [43]	Guatemala	M	37	Liposuction, fat grafting	Yes	Yes	Alive
2021, Kadar A [13]	US	F	26	Liposuction	No	Yes	Alive
2022, Fonseca EKUN [44]	Brazil	F	32	Liposuction	Unknown	Unknown	Unknown
2022, Foula AS [45]	Egypt	F	29	Liposuction	Yes	Yes	Alive
2022, Pham MQ [46]	Vietnam	F	37	Liposuction	No	No	Alive
2022, Wolfe EM-1 [47]	US	F	28	Liposuction, fat grafting	No	Yes	Alive
2022, Wolfe EM-2 [47]	US	F	26	Liposuction, fat grafting	No	Yes	Alive

* The citation numbers in Table 1 revised are the same as in Table 1 of the article.
